# Genome variability of human adenovirus type 8 causing epidemic keratoconjunctivitis during 1986-2003 in Japan

**Published:** 2011-11-30

**Authors:** Xue-Hai Jin, Koki Aoki, Nobuyoshi Kitaichi, Toshihide Ariga, Susumu Ishida, Shigeaki Ohno

**Affiliations:** 1 Department of Ophthalmology, Hokkaido University Graduate School of Medicine, Sapporo, Japan; 2 Department of Ocular Inflammation and Immunology, Hokkaido University Graduate School of Medicine, Sapporo, Japan; 3 Department of Ophthalmology, Health Sciences University of Hokkaido, Sapporo, Japan

## Abstract

**Purpose:**

Epidemic keratoconjunctivitis (EKC) is a contagious acute conjunctivitis associated with community-acquired infection. Human adenovirus type 8 (HAdV-8) is one of the major serotypes isolated from patients with EKC. DNA restriction enzyme analyses were performed to investigate the genetic characteristics of the isolates and their chronological pattern.

**Methods:**

Viral samples were taken from 11 strains isolated from sporadic cases of EKC and identified as HAdV-8 by the neutralization method with type-specific antiserum against HAdV-8 between 1986 and 2003 in Japan. DNA restriction enzyme analysis included six restriction enzymes: BamHI, HindIII, PstI, SacI, SalI, and SmaI.

**Results:**

The restriction patterns revealed that the genome types were HAdV-8A and HAdV-8B in 1986, HAdV-8K in 1991, and HAdV-8E in 1996. HAdV-8K was a new genome type revealed with the enzyme SacI. Two strains isolated in 2003 exhibited identical restriction patterns as HAdV-54, which was described in 2008 and collected from Japanese patients in 2000.

**Conclusions:**

Genetic changes might occur chronologically in HAdV-8. HAdV-8 displays considerable variability. The investigations of these variants might be helpful for defining the evolutionary tendency and to predict future outbreaks of HAdV infection.

## Introduction

Human adenoviruses (HAdVs) cause ocular infections. The most severe disease among ocular infections is epidemic keratoconjunctivitis (EKC), which is characterized by bilateral, acute, severe keratoconjunctivitis and known for frequent intrafamilial infection [1]. EKC is commonly caused by HAdV-8, followed by HAdV-19 and HAdV-37, members of species D of human adenovirus [2,3]. HAdV-8 was first described in the United States in 1955, and the virus was isolated from a sailor (Trim) who had EKC and had arrived from the Orient [4]. Since that time, HAdV-8 has been isolated all over the world from typical cases of EKC. Using restriction enzyme analysis, the serotypes are subclassified into genome types, nominated according to the chronology reported in the literature. HAdV-8A and HAdV-8B were shown to have been circulating in the population of Sapporo, Japan, between 1975 and 1981 [5]. HAdV-8C, D, E, F, G, and H were detected in Kaohsiung, Taiwan from 1980 to 1994 [2,6,7]. The genome type HAdV-8E was also found in South Korea [8]. In Australia and the Philippines, only the prototype strain of HAdV-8 was found [9]. HAdV-8I was isolated from an outbreak of EKC in 1995 and from sporadic cases until 1997 in Hiroshima, Japan [10]. In Europe, HAdV-8 strains isolated in Germany were classified HAdV-8/D1 to HAdV-8/D6, and substitution of the fastidious Trim strain by the well growing strain D1 as a prototype was suggested [11]. Later, additional genome types HAdV-8/D7 to HAdV-8/D10 were reported [12]. Following this nomenclature system, genome types HAdV-8/D11 and HAdV-8/D12 were isolated in Brazil [13].

To date, HAdV-8A, B, E, and I have been found in Japan as variants of HAdV-8. Recently, two novel HAdV types causing nosocomial EKC were reported from Japan [14-16]. One of them has sometimes been mistyped as HAdV-8, because it is similar to HAdV-8 according to neutralization test (NT) and phylogenetic analyses. However, the virus showed completely different restriction patterns from those of other published HAdV-8 genome types, revealing it is a novel serotype. It is named as HAdV-54 today [14].

In the present study, using HAdV-8 strains isolated between 1986 and 2003 in Japan, we reconfirmed the HAdV type by NT and phylogeny-based classification of partial hexon sequences. Moreover, the genetic differences among the isolates were analyzed by DNA restriction enzyme analysis.

## Methods

### Viral strains

Eleven strains of HAdV-8 were isolated from sporadic cases of EKC in Japan (Table 1). Strains number 1, 2, and 3 were isolated in 1986, number 4, 5, and 6 in 1991, and number 7, 8, and 9 in 1996 in Sapporo, northern part of Japan. number 10 was isolated in 2003 in Itoman, the Okinawa region, and number 11 was isolated in 2003 in Matsuyama, both are southweste area of Japan. All isolates were propagated in A549 cells and identified as HAdV-8 using NT. The HAdV-8 prototype strain was purchased from the American Type Culture Collection (Manassas, VA).

**Table 1:** Summary of genome type of 11 HAdV strains isolated in Japan during 1986–2003.

** **	** **	** **	Enzyme code*						** **	** **
**Strains of samples**	**Genome types**	**Years collected**	**BamHI**	**HindIII**	**PstI**	**SacI**	**SalI**	**SmaI**	**Neutralization test titer**	**Samples collected in**
** **	HAdV-8P	1955	1	1	1	1	1	1	>64	(ATCC)
1	HAdV-8A	1986	2	2	2	2	2	2	>64	Sapporo
2,3	HAdV-8B	1986	2	2	2	2	3	2	>64	Sapporo
4,5,6	HAdV-8K	1991	2	3	2	3	2	2	>64	Sapporo
7,8,9	HAdV-8E	1996	2	3	2	2	2	2	>64	Sapporo
10	HAdV-54	2003	3	4	3	4	4	3	32	Itoman
11	HAdV-54	2003	3	4	3	4	4	3	16	Matsuyama

*Enzyme codes are displayed in alphabetical order: BamHI, HindIII, PstI, SacI, SalI, SmaI. The restriction patterns of HAdV-8P for each enzyme are called number 1. The other patterns are consecutively numbered in chronological order of appearance. The numbering in this study was performed according to the methods of previous report [25]. HAdV-8P: HAdV-8 prototype.

### Serological analysis

Those 11 samples of strains were serologically analyzed by a quantitative serum NT with HAdV-8 type-specific antisera purchased from Denka Seiken Co., Ltd. (Tokyo, Japan) to confirm our previous classification. NT was performed in A549 cells on 96-well microplates. The 50% tissue culture infective dose (TCID50) of each HAdV that caused a cytopathic effect after 7 days of incubation at 37 °C was calculated, and 100 TCID50s was used for the challenge virus. Duplicates of the serially twofold diluted antisera were used in the NT.

### Virus propagation and DNA extraction

All of the strains were inoculated into culture tubes containing a subconfluent monolayer of A549 cells. The inoculated tubes were maintained for 1 h at 35 °C for viral absorption, and then 2 ml of maintenance medium was added. The inoculated cultures were incubated at 35 °C with medium changes at intervals of 3 or 4 days and examined daily until the appearance of cytopathic effects. The viral DNA was extracted following a previously described protocol with some modifications [17]. The cells were pelleted and rinsed twice with phosphate-buffered saline and then suspended in 1 ml of Hirt lysis solution (10 mM Tris, 1 mM EDTA, 0.6% SDS, pH 8.0). Proteinase K was added to a final concentration of 50 μg/ml and the samples incubated at 37 °C for 1 h. Cellular DNA was precipitated with NaCl (1 M) overnight at 4 °C and discharged. The supernatant was cleaned with a mixture of RNases A and T1, proteinase K (200 μg/ml), and phenol-chloroform extraction. Viral DNA was precipitated with ethanol and suspended in 50 μl of TE buffer (1 mM Tris-HCl, 0.1 mM EDTA, pH 8.0).

### Phylogeny-based classification for HAdV typing

For HAdV typing, nucleotide sequences in the partial hexon were amplified and subjected to phylogenetic analysis as described previously [18]. The nucleotide sequences of the PCR products were determined using a CEQ 2000XL DNA analysis system with a DyeTerminator cycle sequencing kit (Beckman Coulter, Fullerton, CA) and compared with those of all HAdV types using SINCA (Fujitsu Limited, Tokyo, Japan). The evolutionary distances were estimated using Kimura’s two-parameter method [19], and unrooted phylogenetic trees were constructed using the neighbor-joining method [20]. Bootstrap analyses were performed with 1,000 resamplings of the data sets.

### DNA restriction enzyme analysis

Aliquots of viral DNA (2 μl, approximately 1 μg) were digested with 10 units of restriction enzymes BamHI, HindIII, PstI, SacI, SalI, and SmaI under conditions specified by the manufacturer (Takara Shuzo Co., Kyoto, Japan). Restriction enzyme digests were loaded onto 1.5% agarose gels and run for approximately 2 h at 100 V in Tris-acetate buffer (pH 8.0) with 1 mM EDTA. After staining with ethidium bromide (1 μg/ml), the fragments were visualized under UV transilluminator and photographed with a PolaroidTM camera. Subgenus and genome type identification was performed by comparing the resulting patterns with the published restriction patterns of the prototypes and genome types.

## Results

### Serological analysis

Eleven strains of samples were specifically neutralized with the antiserum against HAdV-8. However, strains of number 10 and 11 isolated in 2003 reacted to HAdV-8 antiserum only at 1:32 and 1:16 of homologous titer respectively, a weak reaction for the immunological distinctiveness of serotype (Table 1). No other prototype-specific antiserums reacted with these 2 strains. These strains of number 10 and 11 collected in Itoman and Matsuyama were considered as one of the HAdV-8s at that time.

### HAdV typing of phylogeny-based classification

The nucleotide sequences of the partial hexon gene were compared with those of all HAdV prototype strains. Strains of number 1 to 9 were clustered in HAdV-8, however number 10 and 11were in HAdV-54 (data not shown). Strains of number 10 and 11 were reclassified as HAdV-54.

### DNA restriction enzyme analysis

**Figure 1:**
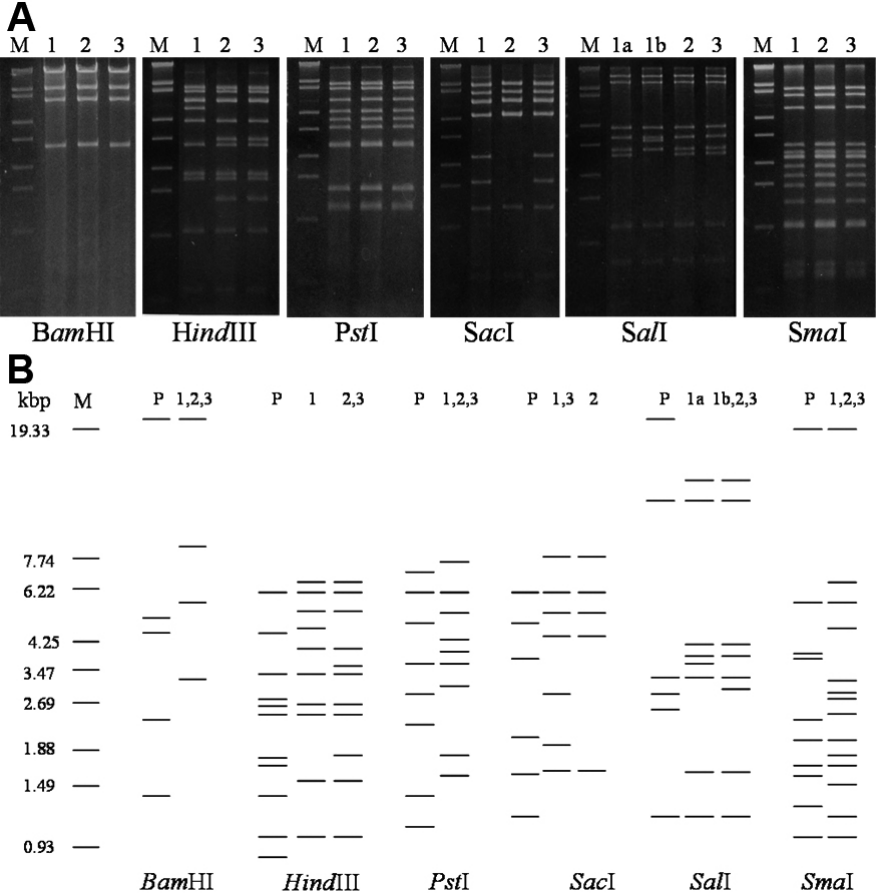
Restriction patterns of strains number 1–9. **A**: Restriction patterns of 9 strains isolated in 1986, 1991, and 1996. Lane M: molecular weight marker (lambda DNA digested with EcoT14I). Lane 1: representative strains number 1–3 isolated in 1986. Lanes 1a and 1b represent the two kinds of restriction patterns for SalI (HAdV-8A and HAdV-8B). Lane 2: representative strains number 4–6 isolated in 1991 (HAdV-8K). Lane 3: representative strains number 7–9 isolated in 1996 (HAdV-8E). **B**: Schematic presentation of the restriction patterns of strains number 1–9 and HAdV-8 prototype.

**Figure 2:**
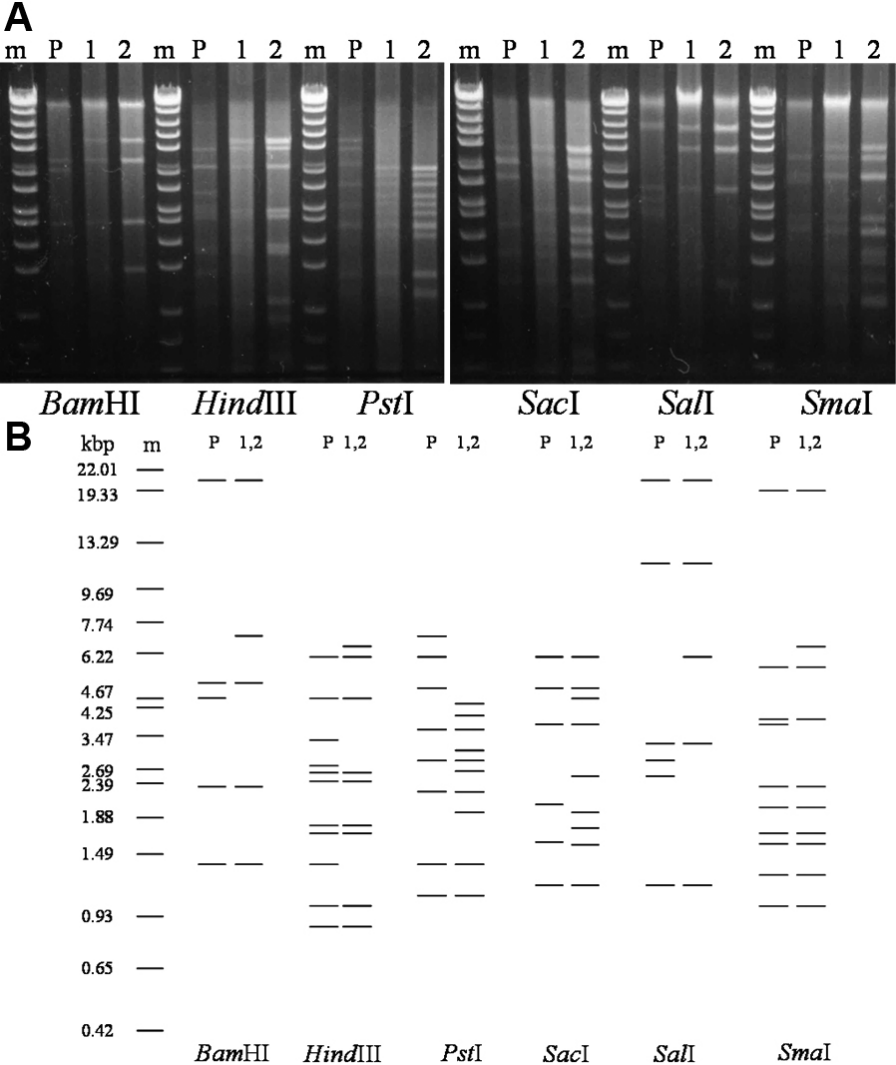
Restriction patterns of strains number 10 and 11. **A**: Restriction patterns of 2 strains isolated in 2003. Lane m: molecular weight marker (lambda DNA digested with EcoT14I plus BglII). Lane P: HAdV-8 prototype. Lane 1: representative strains number 10 isolated in Itoman in 2003 (HAdV-54). Lane 2: representative strains number 11 isolated in Matsuyama in 2003 (HAdV-54). **B**: Schematic presentation of the restriction patterns of strains HAdV-8 prototype and samples number 10 and 11.

After restriction enzyme digestion, the 11 strains were electrophoresed in two gels. Strains of number 1 to 9 isolated in 1986, 1991, and 1996 that reacted strongly to HAdV-8 antiserum were run in one gel (Figure 1A). The remaining 2 strains of number 10 and 11 isolated in 2003 showing weak reaction to HAdV-8 antiserum were run in another gel (Figure 2A). To make the restriction patterns of these strains are easy to read, they were also shown as schematic presentations (Figure 1B and Figure 2B). It was revealed that the 11 strains analyzed in this study were divided into five genome types according to the profile of restriction patterns (Table 1). All of 11 strains showed different restriction patterns from those of the HAdV-8 prototype. Of the 3 strains isolated in 1986, number 1 showed restriction patterns similar to HAdV-8A and number 2 and 3 were similar to HAdV-8B. number 4 to 6 isolated in 1991, and number 7 to 9 isolated in 1996 showed identical restriction patterns with restriction endonucleases BamHI, HindIII, PstI, SalI, and SmaI. With SacI, the strains number 4 to 6 isolated in 1991 exhibited different patterns from those of the strains isolated in 1996 and any other previously known HAdV-8 genome types. This new pattern revealed a new genome type of HAdV-8 that we designated as HAdV-8K in accordance with the serial studies in the Asia Pacific area. The restriction patterns of strains number 7 to 9 isolated in 1996 corresponded to HAdV-8E. The restriction patterns of strains number 10 and 11 isolated in 2003 were similar to those of HAdV-54. The results of DNA restriction enzyme analysis are summarized in Table 1.

## Discussion

To investigate the genetic characteristics of HAdV-8 isolates and their chronological pattern, we demonstrated the molecular biologic characteristics of 11 HAdV strains isolated from sporadic cases of EKC in Japan over an 18-year period in the present study. All of the strains were identified as HAdV-8 by the neutralization method. In the present study, a new variant strain, HAdV-8K, was identified by the DNA restriction method. The DNA restriction method for genome typing identified two strains isolated in 2003 as HAdV-54, not HAdV-8. These were also confirmed as HAdV-54 by phylogenetic analysis of partial hexon gene. HAdV-54 was not detected before the early 1990s, but replaced HAdV-8 in Japan in the 2000s [21]. HAdV-54 in samples collected in 2003 is consistent with that study. However, major causes of EKC vary in different countries, and HAdV-8 is still the most common pathogenic virus in the Middle East [22].

As HAdV-8 has a much higher tropism for conjunctival cells and produces more severe clinical manifestations and pathological alterations in EKC than HAdV-19 or HAdV-37, HAdV-8 has been the target of extensive study [10]. After HAdV-8 was first isolated as an etiology of EKC, many HAdV-8 genome types have been identified by applying restriction endonucleases and cleavage pattern analysis [7]. Distinct nomenclature systems have been used for these genome types. A nomenclature system using a numerical code to denote adenovirus genome types has been proposed, and HAdV-8/D1 to HAdV-8/D12 have been described depending on the chronological order of the respective isolates [11-13]. Another nomenclature system was also proposed in which HAdV-8 genome types are denoted by alphabetical order [5]. According to the nomenclature system, HAdV-8 genome types are classified as HAdV-8A to HAdV-8K [5-7,10,23]. Although both systems are commonly used, we adopted the latter nomenclature system here in accordance with a series of collaborative studies of HAdV-8. We compared the restriction patterns of the strains in this study with those of all previously known genome types of HAdV-8. One new genome type was discovered and designated as HAdV-8K.

The cleavage patterns of 2 strains isolated in 2003 with any of the 6 restriction endonucleases were different from those of any other previously known genome type of HAdV-8. The unique restriction patterns indicated that an uncommon mutation or recombination event might have occurred. The restriction patterns of these 2 strains were consistent with those of HAdV-54, which was first reported in 2008 [14]. Furthermore, these strains were neutralized weakly by the antiserum against HAdV-8. Similarly, HAdV-54 responds weakly to the antiserum for HAdV-8. Because of the absence of serotype specific antiserum, the weak reaction with the antiserum against HAdV-8 might be a clue for detecting HAdV-54 at the current time. The strains of number 10 and 11 were also confirmed as HAdV-54 by phylogeny-based classification, which is an effective tool for the rapid identification of HAdVs.

Although HAdV-8 seems to display considerable variability and adenoviral ocular infection is common in East Asian countries, few studies on the circulation of HAdV-8 genome types have been reported in Japan. Thus, this is a report even more focused on the genetic characteristics of HAdV-8 isolates and their chronological patterns than the analysis of a new genome type. Two studies reported the isolation of HAdV-8A and HAdV-8B in Sapporo [5], and the circulation of HAdV-8 genome types in Hiroshima over a 15-year period [10]. In this study, restriction endonuclease analyses revealed a new genome type, HAdV-8K, in Sapporo, where HAdV-8A and HAdV-8B are endemic. The strains isolated in 1986 were HAdV-8A and HAdV-8B, and HAdV-8K strains were isolated from samples collected in 1991. The strains isolated in 1996 exhibited a similar restriction pattern as that of HAdV-8E, which was first isolated in Kaohsiung, Taiwan [6]. HAdV-8 was reported as being prevalent in 1986, 1991, and 1996 in Sapporo [2,24]. Our present results show that different genome types of HAdV-8 appeared in the three prevalence periods, which may indicate that a change in genome type resulted in the next prevalence period of HAdV-8. On the other hand, high frequency of infection in the population is regarded as a probable reason for the diverse evolution of HAdV-8 [7]. As HAdV-8 has become less common in Japan since 1997, a follow-up study of HAdV-8 should be completed in Japan and why we conducted this study.

HAdV-54 has recently been the major cause of EKC in Japan. The strain has occasionally been identified as HAdV-8 due to its weak reaction to HAdV-8 antiserum. On the basis of the nucleotide sequence identities and phylogenetic analysis, HAdV-54 was much closer to the HAdV-8 strains over the entire genome than other HAdVs [15].

Our results show that genetic changes in HAdV-8 occur chronologically. HAdV-8 displays considerable variability. Because continued investigations of these variants might be helpful for defining the evolutionary tendency and to predict future outbreaks of HAdV infection, further genetic, epidemiological, and clinical surveillance of HAdVs should be performed.
